# Differential Gene Expression in Human Upper Respiratory Tract Samples Identifies Antiviral Responses in Omicron SARS-CoV-2 Infection

**DOI:** 10.3390/genes17050497

**Published:** 2026-04-22

**Authors:** Andrea E. Luquette, Anthony Cicalo, Maren C. Fitzpatrick, Ghyssella E. Valdiviezo, J. Alexander Chitty, Gregory K. Rice, Regina Z. Cer, Cameron V. Sayer, Francisco Malagon, Kimberly A. Bishop-Lilly

**Affiliations:** 1 Genomics and Bioinformatics Department, Biological Defense Research Directorate, Naval Medical Research Command-Frederick, Fort Detrick, MD 21702, USA; 2 Leidos, Reston, VA 20190, USA

**Keywords:** SARS-CoV-2, differential gene expression, nasopharyngeal swab, host pathways, respiratory virus

## Abstract

**Background/Objectives**: SARS-CoV-2 is the causative agent of COVID-19, an infectious viral respiratory disease with human-to-human transmission. Current molecular understanding of how hosts respond to infection by respiratory viral pathogens in general and to SARS-CoV-2 in particular is still a research field under development. The activation levels of various host pathways are dependent on several variables, including the host tissue compartment. **Methods**: In this work, Illumina RNA sequencing was performed to assess the transcriptional host response to SARS-CoV-2 infection using COVID-19 PCR testing nasopharyngeal (NP) swab remnants from twenty infected and nine non-infected individuals. **Results**: Differential gene expression (DGE) analysis identified 182 overexpressed genes, with strong enrichment in innate immune and viral response genes. This included a significant induction of *IFIH1/MDA5*, a pattern recognition receptor (PRR) gene participating in the initial sensing of viral RNAs and subsequent cascade activation of interferon (IFN) and IFN-stimulated genes (ISGs). Interestingly, we observed different levels of concordance with previous similar studies and a significant induction of *RIG1* and *TLR3*, two PRR genes encoding proteins that function to upregulate IFN and ISGs, but which are not normally identified as differentially expressed genes (DEGs). Finally, the overexpression of *MX1*, a well-characterized biomarker of viral infection; *IFIT1*, one of the top upregulated genes; and *OAS1*, *OAS2* and *OAS3*, genes with a molecular function, 2-5-oligoadenylate synthase activity, identified as enriched in the DGE analyses, was confirmed by RT-qPCR. **Conclusions**: This study provides insights into upper respiratory tract responses to SARS-CoV-2 infections and identifies a set of differentially expressed genes (DEGs) with potential as candidates for further investigations as viral infection biomarkers.

## 1. Introduction

The respiratory tract is one of the most common entry points for invading pathogens as well as a major route for human-to-human transmission. As such, evolution has favored robust local adaptive and innate immune responses to prevent infection and to fight pathogens that propagate in this area of the body. Notwithstanding individual and social immunity developed to protect from respiratory infections [1,2], respiratory pathogens constitute a major class of microbes behind seasonal illnesses, epidemics and pandemics [3,4]. In contrast to adaptive immunity, which is long-lasting, highly pathogen-specific, and slow to develop, innate immune cellular responses are rapid, short-lived, and nonspecific, in that even though there is some degree of variability of the cellular responses depending on the pathogen, innate immune responses are considered broad-ranged. This is achieved starting at the pathogen recognition level, where there are sets of sensors that recognize molecular signatures typical of broad classification groups. An example of this would be the Toll-like receptors (TLRs) [5,6], with TLR3 recognizing viral RNA and TLR4 acting as a sensor for Gram-negative bacterial lipopolysaccharide. These sensors activate the transcription of interferon genes that, in turn, lead to the JAK-STAT-mediated upregulation of a multitude of interferon-stimulated genes (ISGs) involved in secondary sensing of viral components, inflammatory responses, and antiviral resistance [7]. Therefore, different degrees of pathogen class specificity could be inferred from the specific set of innate immune genes activated by different types of pathogens, and, in turn, this knowledge could be used to improve diagnosis and prognosis.

Severe acute respiratory syndrome coronavirus-2 (SARS-CoV-2), with the official name *Betacoronavirus pandemicum* [8], is a positive-strand RNA virus of the subgenus *Sarbecovirus* genus *Betacoronavirus*, and the causative agent of COVID-19, an infectious viral disease that emerged in 2019 and resulted in a global pandemic from 2020 until 2023 and seasonal illness afterwards [9]. Respiratory viruses with very different genomic organization and epidemiologic characteristics, like SARS-CoV-2, influenza virus and others, have been subject to extensive research due to high burden for healthcare systems, including studies on transcriptional host responses and on the identification of tissue-specific infection biomarkers [10,11,12,13]. Crowded environments, typical, for example, of military facilities and ships, favor the spread of respiratory viruses that could result in outbreaks if robust early-warning surveillance protocols are not available. Therefore, the identification of host biological pathways and individual genes upregulated in SARS-CoV-2 infected individuals could provide valuable biomarker information to be used in design of diagnostic assays to support force health protection decision-making during the occurrence of respiratory illness outbreaks.

To increase the reliability of previously identified DEGs in response to SARS-CoV-2 infection and to find new putative DEGs, we conducted RNA sequencing transcriptomic analysis of residual nasopharyngeal (NP) swab samples from SARS-CoV-2-positive versus SARS-CoV-2-negative individuals. These data could not only be applied to gain further insight into cellular pathways activated by SARS-CoV-2 in the respiratory tract, but also to identify potential biomarkers with different degrees of pathogen specificity as candidates for further clinical evaluation.

## 2. Materials and Methods

### 2.1. Sample Source

The biospecimens for this work were commercially sourced (iSpecimen, Woburn, MA, USA) and consisted of deidentified residual NP swabs in the universal transport media (Becton, Dickinson and Company, Franklin Lakes, NJ, USA), collected from individuals tested for COVID-19 by RT-qPCR using GeneXpert systems (Cepheid, Sunnyvale, CA, USA) (Table 1) and with no known viral coinfections. Upon receipt, viral infection status was confirmed internally via sequencing and bioinformatics, and five samples were excluded due to either failing the library preparation step or producing very few SARS-CoV-2 sequencing reads upon SARS-CoV-2 amplicon sequencing, as in our experience, both of those outcomes can indicate samples containing very little virus. In addition, among the samples that passed those prerequisites, one of the COVID-19-negative samples, sample NP71, was excluded from the DGE analyses due to the detection of human parainfluenza virus 1 in the sample after sequencing and analyses with VirusSeeker 2.0 [14], leaving nine SARS-CoV-2-negative samples for use in downstream analyses. This study was designated as non-human subject research by the Naval Medical Research Command Institutional Review Board with approval number PJT-23-07 DoHSR_ORA.

### 2.2. RNA Extraction

Total RNA was extracted from 250 μL of residual viral transport medium (VTM) from fifty-five (although only twenty-nine of them passed all subsequent quality controls for inclusion in this study; see Table 1 and Section 3) NP swab samples using Trizol LS reagent (Thermo Fisher, Waltham, MA, USA) using the manufacturer’s protocol. Briefly, the samples were incubated with 750 μL of Trizol LS for 10 min, followed by the addition of 200 μL of chloroform, inversion of the tubes, and centrifugation to separate the phases. The top aqueous phase was recovered into a new tube and precipitated with 0.9 volumes of isopropanol after 1 h incubation on ice and centrifugation at 20,000 rpm for 15 min. The supernatant was discarded, and the pellet was washed with 75% ethanol. The cleaned RNA was eluted in 35 μL of molecular biology-grade water (Thermo Fisher). Quality control of RNA samples was conducted using Qubit RNA quantification kits and a Qubit 3 fluorometer (Thermo Fisher) and/or a high-sensitivity RNA TapeStation kit and TapeStation 4150 electrophoresis system (Agilent, Santa Clara, CA, USA).

RNA for RT-qPCR was obtained by first extracting total nucleic acid (TNA) from 200 μL of residual VTM from NP swabs using the QIAamp DNA Blood Mini kit (Qiagen, Germantown, MD, USA) using the vendor’s recommended protocol, with the exception that no RNAase was added to the reagents. The TNA was then treated with HL-dsDNAse (Qiagen) by mixing 50 μL of TNA, 1 μL of NEB restriction buffer 2, and 2.5 μL of HL-dsDNAse, and incubating at 37 °C for 10 min followed by 52 °C for 15 min. We used this second RNA extraction method due to an improved efficiency of recovery of RNA and lower Ct values for RT-qPCR compared to the Trizol LS method.

### 2.3. SARS-CoV-2 Amplicon Library Preparation and Sequencing

SARS-CoV-2 amplicon library preparation and DNA sequencing were performed as previously described with small modifications [15]. Briefly, 8 μL of total RNA was used as input for the NEB ARTIC library prep kit (New England Biolabs, Ipswich, MA, USA) following the manufacturer’s protocol, with the exception that ARTICv4.1 primers [16] were used instead of the amplification primers provided with the kit. The libraries were assessed for quality using DNA 1000 (Agilent) and Qubit dsDNA BR Assay kits (Thermo Fisher), pooled, and sequenced using a MiSeq sequencing system and a MiSeq v3 600 cycles reagent kit (Illumina, San Diego, CA, USA).

### 2.4. Shotgun RNA Library Preparation and Sequencing

Shotgun RNA sequencing library preparation and sequencing were performed as previously described with small modifications [17]. Briefly, 5 μL of total RNA was used as input for the NEBNext Ultra II RNA Library Prep Kit (New England Biolabs) following the manufacturer’s instructions. The libraries were assessed for quality using D1000 ScreenTape and D1000 reagents (Agilent) and Qubit dsDNA BR Assay Kits (Thermo Fisher), pooled, and sequenced using a NovaSeq 6000 sequencing system and an S1 v1.5 300 cycles reagent kit (Illumina).

### 2.5. Genomic Analyses of SARS-CoV-2 Amplicon Sequencing Data

Amplicon sequencing reads were processed using Viral Amplicon Illumina Workflow (VAIW) [18]. Sequencing reads were trimmed, filtered to Q20 and a minimum length of 50 bp, merged with default settings and aligned to the Wuhan reference genome (GenBank accession NC_045512.2) with local alignment and a maximum insertion/deletion of 500 bp using BBTools. SARS-CoV-2 lineage determination was carried out using Pangolin (Phylogenetic Assignment of Named Global Outbreak LINeages) v.4.3.1 [19] and Constellations repository v0.1.12 [20].

### 2.6. Identification of Sequencing Reads from Pathogenic Viruses

Raw shotgun sequencing reads were screened for the presence of human viral pathogens using VirusSeeker 2.0 [14], an enhanced version of VirusSeeker [21]. Briefly, quality-controlled sequencing reads were assembled into contigs using metaSPAdes [22], then contigs and stitched reads were used for alignment nucleotide identity BLASTN 2.17.0 searches against databases restricted to human pathogenic viruses.

### 2.7. Bioinformatic Analysis of Total RNA Sequencing Data

Shotgun sequencing reads were processed following the nf-core RNA-Seq v3.17.0 pipeline [23], and the workflow was managed using Nextflow v24.04.2 [24]. Read quality was assessed using FastQC v0.12.1, trimmed and quality filtered with fastp v0.23.4, and ribosomal RNA (rRNA) sequences were removed with sortMeRNA v4.3.7 [25]. The reads were then aligned to the Genome Reference Consortium (GRC) h38 human genome using the splice-aware STAR v2.7.11b aligner [26]. Resulting BAM files were sorted, indexed, and assessed using SAMtools v1.2 [27], and transcript abundance was quantified using Salmon v1.10.3 [28]. Quantification quality control was performed using Qualimap v2.3 [29], dupRadar v1.32.0 [30], and the RSeQC suite (v5.0.2) [31], with results summarized in a MultiQC report. The transcript abundance results were imported into R via tximeta v1.20.1 [32], where DGE analyses were carried out with DESeq2 v1.46.0 [33] applying an adjusted *p*-value ≤ 0.05 and an absolute log2-fold-change ≥ 2. Gene set enrichment analysis (GSEA) were performed in R using clusterProfiler v4.14.0 [34], and online using STRING v12.0 database [35] with default parameters and focus on the Gene Ontology (GO) enrichment analysis tool for functional pathways/biological processes analyses [36].

### 2.8. Quantitative RT-PCR

Gene expression quantifications by RT-qPCR were carried out in triplicate using 3 μL of total RNA, gene-specific TaqMan assays (Thermo Fisher), PrimeTime One-Step RT-qPCR master mix (Integrated DNA Technologies, Coralville, IA, USA) and a CFP Connect qPCR system (BioRad, Hercules, CA, USA) with the following cycling conditions: 50 °C 30 min, 95 °C 3 min, 45 amplification cycles (95 °C 15 s plus 60 °C 1 min). The specific TaqMan assays used were Hs03027069_s1 FAM-MGB, Hs00895608_m1 FAM-MGB, Hs00242943_m1 FAM-MGB, Hs00942643_m1 FAM-MGB, Hs0019632_m1 FAM-MGB, and Hs01124518_m1 VIC-MGB for *IFIT1*, *MX1*, *OAS1*, *OAS2*, *OAS3*, and *RPP30* respectively. Normalized fold changes were calculated using double delta Ct analysis [37].

## 3. Results

### 3.1. Sample RNA Processing and Selection Criteria

Total RNA from NP swabs is one of the most common inputs for nucleic acid amplification tests (NAAT) for SARS-CoV-2 detection [38], and host biomarker assessment for COVID-19 [39] and other respiratory viral infections [10,11]. The samples in this study consist of a heterogeneous set of deidentified remnant NP swabs originally used for COVID-19 PCR testing (Table 1) and divided into two categories: SARS-CoV-2-positive and SARS-CoV-2-negative. Due to the high degree of heterogeneity in the quantity of biological material among the samples available, the following criteria were applied for sample selection for this study: (a) samples with detectable levels of RNA (~1 ng/μL or higher), (b) relatively balanced number of samples from male and females in each of the two categories, (c) include only SARS-CoV-2-positive with a robust level of SARS-CoV-2 (resulting in breadth of coverage > 90% upon amplicon sequencing), and (d) avoid including samples with infections with other respiratory viruses. In addition, we reasoned that instead of using poly(A)^+^ purified RNA, a common preprocessing in transcriptomic studies to eliminate highly abundant stable RNAs like rRNAs, we will use total RNA for library preparation and sequencing due to: (a) the low amount of starting material, and (b) the rarity of using poly(A)^+^ RNA in the clinical diagnostics setting. Using these criteria, 60 samples were selected (45 SARS-CoV-2-positive and 15 SARS-CoV-2-negative) for RNA extraction using Trizol LS, and after quality control, 40 SARS-CoV-2-positive and 10 SARS-CoV-2-negative samples were considered as having enough RNA for the next step.

### 3.2. SARS-CoV-2 Amplicon Sequencing

SARS-CoV-2 detection and genetic characterization were carried out by amplicon sequencing using ~400 bp ARTIC Consortium SARS-CoV-2 amplicons for short read sequencing library preparation as indicated in the Materials and Methods. The libraries were pooled and sequenced on an Illumina MiSeq system, resulting in an average coverage of 1.2 M raw reads per sample. In agreement with the metadata provided, none of the 15 samples classified as SARS-CoV-2-negative by RT-PCR produced amplicon libraries. In addition, four of the samples classified as SARS-CoV-2-positive did not produce amplicon libraries passing the quality control, and therefore were not sequenced, and one resulted in mediocre coverage of the SARS-CoV-2 genome upon sequencing and was therefore excluded from downstream analyses. Moreover, additional samples were omitted because out of the 39 SARS-CoV-2-positive samples producing adequate SARS-CoV-2 amplicon sequencing data, only 30 also produced good total RNA shotgun sequencing libraries (next section). All 30 selected SARS-CoV-2-positive samples resulted in amplicon sequencing data with SARS-CoV-2 breadth of coverage > 96%, suggesting a moderate or high viral load in all of them (Table 1). Variant analyses of the genomes assigned all of them to omicron variants of pangolin lineages, in agreement with the reported circulating variant at the time of collection of the samples (June–July 2022) [9]. Therefore, we confirmed and expanded the RT-PCR information provided in the sample metadata.

### 3.3. RNAseq Shotgun Sequencing: Presence of Pathogenic Viruses

Next, shotgun Illumina libraries were prepared from total RNA by random priming reverse transcription as described in the Materials and Methods. Due to the very low amount of starting material in some of the NP swab samples, some of the libraries did not pass quality control, resulting in a final number of 20 SARS-CoV-2-positive and 10 SARS-CoV-2-negative, all of which were pooled and sequenced on a NovaSeq 6000 system, resulting in an average coverage of 103 M raw reads per sample. Infections with other respiratory viruses could significantly alter the host transcriptome and thus complicate analyses and interpretations. Therefore, after completing the initial quality processing of the sequencing reads, we decided to evaluate the presence of pathogenic viruses in the samples using VirusSeeker 2.0, an analysis pipeline developed and extensively tested by our team [14]. As shown in Table 1, this tool confirmed the SARS-CoV-2-positive result obtained by amplicon sequencing and analysis with VAIW [18] and the initial assessment by the sample provider of no known coinfections with other viruses. However, one of the SARS-CoV-2-negative samples, NP71, contained a high number of human parainfluenza type I virus (HPIV-1) reads and was therefore omitted from further analyses.

### 3.4. Host Biological Pathways Upregulated by SARS-CoV-2 Infection

**Figure 1:**
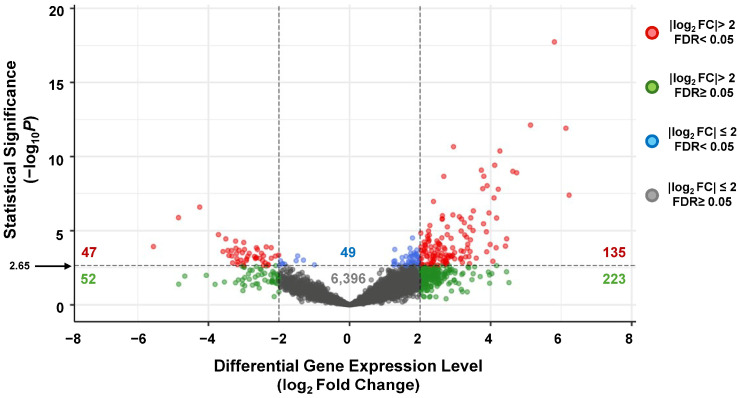
Volcano plot of differentially expressed genes in nasopharyngeal swab samples of SARS-CoV-2-positive versus SARS-CoV-2-negative samples. The value 2.65 on the *y*-axis is indicated with an arrow to highlight the significance cutoff line. Y values > 2.65 have a false discovery rate (FDR) < 0.05 based on the Benjamini–Hochberg *p*-adjusted FDR evaluation method. A total of 6902 genes, with base mean signal ≥ 0.03, are represented in the plot as colored circles located on the XY plane based on their fold changes and statistical significance values. The numbers in the volcano plot correspond to the number of genes in each section of the graph, e.g., 135 are the number of genes considered upregulated (log_2_FC > 2) and statistically significant (FDR < 0.05).

Next, low-count genes were prefiltered before differential expression analysis by requiring counts ≥ 10 in at least three samples, resulting in 6902 genes retained for DESeq2 (Table S1). Among the genes excluded from this work are those with low expression levels in NP swab samples, those producing short RNA transcripts (library molecules corresponding to RNAs of size < 200 nt are eliminated during library preparation) and rRNAs (eliminated during the bioinformatic preprocessing of the sequencing reads). To minimize statistical fluctuations due to low expression, genes with a normalized base mean signal < 0.03 were not included in the analyses. From the total of 6902 genes that passed this filter (Table S1), 4735 (69%) were upregulated and 2167 (31%) downregulated. Classification of DEGs as statistically significant was based on mid/strong fold changes (|log2 FC| > 2.0) and a log_10_ *p*-values cutoff of 2.65, corresponding to false discovery rates (FDR) of 0.05 or lower, considered the standard value for differential gene expression analyses [40]. The final number of statistically significant genes with these criteria was 135 (2.0%) upregulated and 47 (0.7%) downregulated (Figure 1 and Table S2).

**Figure 2:**
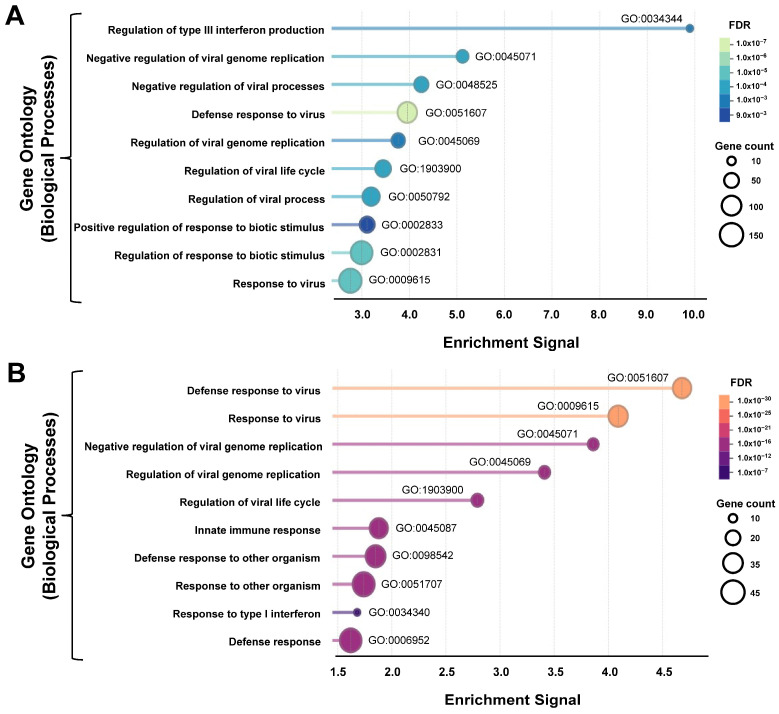
Top ten Gene Ontology (GO) biological processes enriched in NP swab samples of SARS-CoV-2-positive samples. (**A**) GSEAPreranked, including all 4735 genes with no changes or upregulated (log_2_FC ≥ 2). Genes were given ranks according to the formula |log_2_ FC| × −log_10_(*p*-value). (**B**) GSEA including the 135 statistically significant upregulated genes (log_2_FC > 2 and FDR < 0.05). Description of the GO biological processes, GO IDs, FDRs and number of DEGs are indicated in each panel.

Next, we evaluated the enrichment of pre-classified gene sets at the Gene Ontology (GO) database [36], focusing on genes clustered by biological processes. Gene set enrichment analyses (GSEA) and GSEAPreranked, either having into consideration all 4735 upregulated genes (Figure 2A) or only the 135 strongly and significantly upregulated DEGs (Figure 2B), have very low FDRs and a high degree of overlap. In both cases, the top enriched GO processes indicate stimulation of innate immunity genes, including inflammatory responses, negative regulation of viruses, interferon production, and ISGs. The enrichment of these pathways is consistent with viral infection in the SARS-CoV-2-positive samples, further validating the RT-PCR and sequencing data as well as the processing of the samples. This conclusion was further validated using alternative gene sets like the ones in databases such as WikiPathways [41] and UniProt Annotated Keywords [42], with WikiPathways being more precise at pinpointing specifically towards SARS-CoV-2 infection (Figures S1 and S2). By contrast to the upregulated genes, GSEA of downregulated genes did not identify any specific gene sets with any of the databases.

### 3.5. Host Genes Upregulated by SARS-CoV-2 Infection

Next, a more granular investigation of the transcriptomic data was conducted, focusing on individual DEGs. The literature searches on the top twenty upregulated DEGs confirmed the GSEA results by highlighting genes with important functions in broadly innate immunity. Interestingly, although genes involved in general illness responses, e.g., inflammation, were indeed identified, a high proportion of the induced genes could be ascribed to different layers of host defense against invading pathogenic RNA viruses (Table 2).

Some of the genes encoding more general responses to illness are *CXCL9*, *10*, and *11*, producing chemokines, and the inflammation regulator gene *IDO1*. One of the DEGs encoding earlier antiviral infection factors is *RIG1* (Table 2; Fold Change Rank (FCR) 14). The RIG-I protein is a PRR that senses the presence of viral RNA by detecting molecules with 5′ triphosphate and short regions of double-strandedness [58,65,66], but with a modest role in COVID-19 due to its targeted degradation by SARS-CoV-2 [67]. Moreover, *IFIH1/MDA5* and *TLR3*, genes that also encode the first line of defense PRRs, in this case MDA5, a RIG-I-like receptor and the major PRR for SARS-CoV-2 [58,68], and the Toll-like Receptor 3 [63], albeit not in the top 20 (FCRs 24 and 44), are strongly upregulated. Genes participating in host processes downstream of the initial recognition of pathogen-associated molecular patterns (PAMP) and activation of type I IFN genes are among the top upregulated DEGs as well. Those include genes that target a variety of viral life cycle steps to counter virus propagation. *MX1* (FCR 50) encodes MxA, an ancient dynamin-like protein with a broad antiviral spectrum and mode of action not fully understood yet, but possibly involved in virus sequestration [64,69]. *IFITI* (FCR 3), *IFIT2* (FCR 4) and *IFIT3* (FCR 18) inhibit viral translation [45,70], *DDX60L* (FCR 10) inhibits viral replication [51], *RSAD2*/Viperin (FCR 13) counteracts virus life cycles at various levels [54,55,56], and *XAF1* (FCR 6) precludes viral propagation by inducing apoptosis [48]. Other DEGs are involved in the modulation of RIG-I and IFIH1/MDA5 responses. *OASL* (FCR 2) participates in a positive feedback loop that enhances RIG-I activity [44]. *ISG15* (FCR 5) has opposite effects on IFIH1/MDA5 and RIG-I, enhancing the activity of the former and inhibiting the latter [46,47]. *FYB1/ADAP* (FCR 16) counteracts viral evasion factors that suppress RIG-I [59]. Host genes induced by viruses to avoid detection are also included in the list of DEGs. For example, *PNPT1* (FCR 17) counters the host pathway to detect hyperactivity of the mitochondria, typically associated with rapid viral propagation [60]. Additionally, upregulated DEGs clearly linked to viral host responses, e.g., *OAS2*, *TRIM38*, and *STAT2*, but not in the top 20, as well as significantly downregulated genes, are included in Table S2.

### 3.6. Quantification of IFIT1,MX1, OAS1, OAS2 and OAS3 RNAs by RT-qPCR Supports Their Use as Viral Infection Biomarkers

The upregulated DEGs identified in this work contain a high proportion of genes that are clearly involved in host responses to viral infection and/or that have previously been proposed as NP tissue viral infection biomarkers. Among the latter are included *CXCL10*, *IFIT2*, *OASL*, *IFI44L*, and *HERC6*, to name a few [11,12]. Thus, the data presented here could support the use of previously described biomarkers as well as help identify new biomarkers of SARS-CoV-2 infection. Two of them, *IFIT1* and *MX1*, were chosen as proof-of-principle. *IFIT1* is one of the genes with the highest upregulation levels (Table 2) and has been previously proposed as a cancer biomarker [71,72], but, to our knowledge, not for viral respiratory infections. On the other hand, *MX1* has a significantly lower upregulation level than *IFIT1*, but has been used as a blood biomarker for several different respiratory viral infections, including SARS-CoV-2 [13,73]. To strengthen the results obtained by RNASeq, the upregulation of these genes was evaluated using an orthogonal method, specifically a commercially available TaqMan RT-qPCR assay targeting the last exon of the *IFIT1* (Genbank accession NM_001548.5) and the junction between exons 9 and 10 of *MX1* (Genbank accession NM_002462.5). For normalization purposes, a TaqMan RT-qPCR assay targeting the junction between exons 2 and 3 of *RPP30* (Genbank accession NM_006413.4) was used. *RPP30*, encoding the p30 subunit of RNAseP, is commonly used as a housekeeping control gene in SARS-CoV-2 NAAT diagnostics assays of NP swab samples [38] and does not exhibit significant expression changes between SARS-CoV-2-positive and SARS-CoV-2-negative samples (Table 2). Using this approach, the fold upregulation for *IFIT1* and *MX1*, calculated using the *ΔΔ*Ct method, was 20.6 and 4.0-fold respectively. Therefore, although the magnitude of upregulation calculated by sequencing and qPCR is not identical due to multiple different variables, the RT-qPCR results confirm the overexpression of *IFIT1* and *MX1* mRNA in NP samples upon SARS-CoV-2 infection.

**Figure 3:**
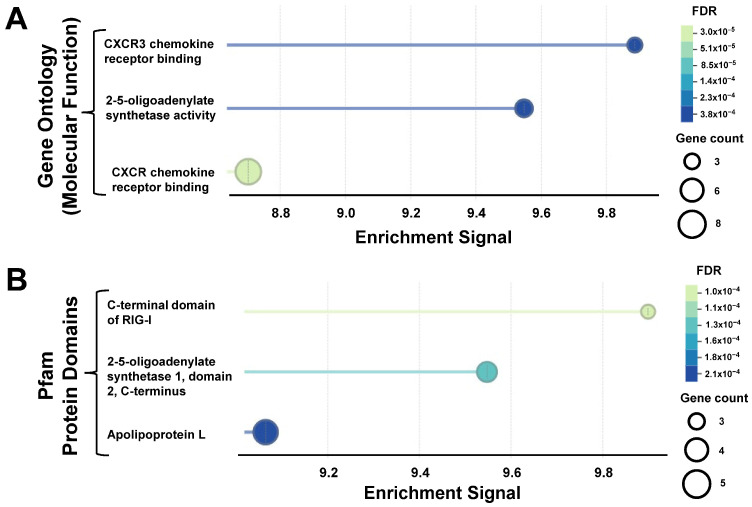
Molecular functions and protein domains enriched in NP swab samples of SARS-CoV-2-positive samples. Gene Ontology molecular functions (**A**) and Pfam protein domains (**B**) enriched, as determined by GSEAPreranked analyses. Includes all 4735 genes with no changes or upregulated (log2 FC ≥ 2). Genes were given ranks according to the formula |log_2_ FC| × log_10_(*p*-value).

Finally, *OAS1*, *OAS2* and *OAS3*, three genes encoding enzymes that synthesize 2′-5′ linked oligoadenylates (2-5A) and that, like in the case of other PRRs, have a strong posttranslational regulation, were selected for further evaluation. Albeit not exhibiting the highest levels of all the upregulated genes in this study, they are known to function as PRRs, and it was notable that pre-ranked GSEA highlighted them when analyzing protein families and enzymatic activities enriched upon SARS-CoV-2 infection (Figure 3).

In addition, these three genes are clustered in locus chr12q24.13, a region in chromosome 12 with genetic alleles associated in other studies with SARS-CoV-2 infection propensity and severity [74,75,76], a fact shared by other DEGs belonging to the innate immune pathway, including the major PRR regulator *IFIH1/MDA5* [68,77]. However, to our knowledge, RNA levels of *OAS1*/*OAS2*/*OAS3* have been previously suggested as candidate biomarkers for other diseases, mainly for cancer, but not for COVID-19 [78,79,80]. As previously, TaqMan RT-qPCR assays and *ΔΔ*Ct values were used as an orthogonal method to assess RNA levels. These assays target the junction between exons 2-3, 1-2, and 3-4, for *OAS1* (Genbank accession NM_001032409.2), *OAS2* (Genbank accession NM_001032731.1) and *OAS3* (Genbank accession NM_006187), respectively. Using this approach, the fold upregulation for *OAS1*, *OAS2* and *OAS3* was found to be 12.8, 4.1 and 15.8 respectively.

These results are in support of the further evaluation of *IFIT1*, *MX1*, *OAS1*, *OAS2* and *OAS3* as NP biomarker candidates for COVID-19. Further studies with time points and a larger number of samples are needed to assess how early these biomarkers are detectable and how durable the signal is over time.

## 4. Discussion

Here we present differential RNA expression data and analyses of NP swab samples from individuals tested for COVID-19, comparing SARS-CoV-2-positive and -negative samples. The initial classification was originally carried out by RT-PCR during routine clinical diagnosis and further confirmed by amplicon and agnostic sequencing. SARS-CoV-2 genomic characterization of the SARS-CoV-2-positive samples indicated that they belong to typical omicron lineages prevalent during the Summer of 2022, e.g., BA.2.12.1 [81]. In addition, we also characterized the samples to ensure that none of the samples included in the differential expression analyses would have been infected by other pathogenic viruses. With regard to the RNA population captured, the RNA processing, sequencing and analyses pipelines used are poly(A)^+^ independent but exclude small RNAs, rRNA genes, and genes with very low expression levels.

DGE analyses of host genes upregulated by SARS-CoV-2 in NP swab samples demonstrated a clear enrichment of GO biological pathways involved in viral infection responses, a conclusion supported by similar analyses using UniProt and WikiPathways databases. The most abundant group of induced genes is ISGs, suggesting that most DEGs are upregulated by transcriptional activation cascades triggered by the recognition of viral PAMPs [6,7,82], although additional mechanisms like increased stability by mRNA pseudouridylation can also play an important role [83,84]. These results, highlighting an interferon-driven response to SARS-CoV-2 Omicron variant infection, are in line with previous studies on NP host responses in pre-Omicron COVID-19 patients [11,12,85] but significantly distinct from other studies where ISG-ranked expressions are significantly lower [86,87]. Interestingly, our data are similar to in vitro studies with cells in culture stimulated by the synthetic RNA SLR14, a potent inducer of RIG-I protein activity [11]. Thus, our data support the use of SLR14 for in vitro studies mimicking NP SARS-CoV-2 infections.

The top overexpressed genes of this study are in common with the top DEGs from two other similar studies where the first line of defense PRR gene *IFIH1/MDA5* as well as the ISGs *CXCL9/10/11*, *IFIT1/2/3*, *OASL*, *RSAD2* and *MX1* top the ranks of DEGs [11,12], but are significantly or sharply ranked lower in other two studies [86,87]. By contrast, *RIG1* and *TLR3* [82] are among the top upregulated genes in our study, but, to our knowledge, not in previous ones. *RIG1* and *TLR3* transcription is generally low and tightly regulated, with *RIG1* being significantly induced by all-trans retinoic acid (ATRA) [57], and in ovarian cancer [88], and *TLR3* during dendritic cell differentiation [89]. However, in most cases, their viral responses are post-translationally regulated by their enzymatic activation upon binding viral RNAs. Other top DEGs from this work that normally do not appear in other similar studies are *AQP9* (FCR 7), an aquaporin with an important role in immune cell activation [49,90], and *PNPT1* (FCR 17), an RNAse commonly induced by viruses to attenuate the host integrate stress response [60].

As expected by the conservation of the ISG response as an antiviral defense, most of the top ISGs in this study are also induced by other respiratory viruses [12]. Specifically, the highlighted ISGs above, *CXCL9/10/11*, *IFIT1/2/3*, *OASL*, *RSAD2*, *MX1*, as well as *ISG15*, are habitually among the top induced genes and, therefore, have the potential to serve as candidates for further clinical testing to determine their suitability as NP biomarkers for the detection of respiratory virus infections. Certainly, a good proportion of these ten genes have already been studied with this specific purpose, with *CXCL10* (FCR 15) being perhaps the most widely used [10,11,43]. A caveat of this study is that the heterogeneity of the samples regarding donor backgrounds and vital statistics may influence the basal and RNA induction levels of specific genes in a differential manner. For example, *IFIH1/MDA5* expression changes significantly with age, an effect that is also modulated by the genotypes of each individual [68,91,92], and environmental conditions affecting the gut microbiota regulate interferon responses to viral infections [93]. Furthermore, the limited amount of sample metadata precludes more in-depth analysis of association with common clinical biomarkers. Complementary studies with carefully controlled sample sources are necessary to address these limitations. However, even with all the limitations indicated above, the results reinforce a strong antiviral host response, albeit with few SARS-CoV-2-exclusive signatures.

We initially chose *IFIT1* and *MX1* as two alternative genes with different ranks (FCRs 3 and 50 respectively), and levels of induction for validation by RT-qPCR, and both showed significant induction using this orthogonal method. In addition, the *OAS1/OAS2/OAS3* genes, exhibiting a significantly more modest fold induction based on the RNA-Seq data (~4–5-fold; see Tables S1 and S2), also exhibit a robust increase in RNA levels in NP swab samples of COVID-19 cases as measured by RT-qPCR. It would be interesting to extend these studies to other respiratory viruses to delimit their specificity.

The upregulated host genes described here, either individually or as a subset in combination, could be used as the starting point to assess their suitability as part of rapid qRT-PCR diagnosis of respiratory viral infection using NP swabbing, or even less invasive sampling methods (e.g., saliva collection). Moreover, the specific combination of enrichment genes in our set could support the diagnosis of illnesses caused by novel or uncommon respiratory viruses. Interestingly, a significant proportion of the DEGs identified here also constitute part of a set of “core response” human genes upregulated in saliva samples upon infection by a broad range of pathogens (e.g., *MX1* and *ISG15*) [94]. Subsequent studies with non-core response genes, e.g., *TLR3*, and using saliva samples could help further facilitate the design of systems specific to respiratory virus infection that could be used with sampling methods even less invasive than NP swabs. Finally, the data herein, when viewed in the light of other similar studies, might suggest that the strong interferon response, ISG upregulation, and PRR activation within the host are a natural means to fight SARS-CoV-2 that could serve as inspiration for studies using drugs mimicking those effects for prophylactic purposes during viral outbreaks.

## Figures and Tables

**Table 1:** Sample metadata and pathogenic virus identification by sequencing.

Sample ID	Metadata				Amplicon Sequencing		Shotgun Sequencing
	Age	Sex	Diagnosis	PCR *	Breath of Coverage ^#^ (%)	Lineage ^@^	Virus ID ^
**NP27**	67	F	COVID-19	+	99.1	BF.5	SARS-CoV-2
**NP28**	3	M	COVID-19	+	100.0	BA.4.1	SARS-CoV-2
**NP30**	2	M	Croup	+	100.0	BA.4.1	SARS-CoV-2
**NP31**	78	F	COVID-19	+	100.0	BA.5.2.9	SARS-CoV-2
**NP34**	77	M	COVID-19	+	99.1	BF.5	SARS-CoV-2
**NP37**	71	M	Sepsis, COVID-19, unspecified altered mental status	+	99.2	BA.2.12.1	SARS-CoV-2
**NP38**	64	M	COVID-19	+	100.0	BA.2.12.1	SARS-CoV-2
**NP43**	92	F	Nausea, vomiting, diarrhea, COVID-19	+	100.0	BF.10	SARS-CoV-2
**NP45**	5	F	COVID-19	+	100.0	BA.2.12.1	SARS-CoV-2
**NP46**	75	M	Other chest pain	+	99.2	BA.5.2.1	SARS-CoV-2
**NP48**	75	F	COVID-19	+	100.0	BA.2.48	SARS-CoV-2
**NP51**	18	M	COVID-19	+	100.0	BA.5.1	SARS-CoV-2
**NP54**	45	F	Weakness, abscess of the left foot, acute kidney injury, sepsis	+	96.6	BG.5	SARS-CoV-2
**NP55**	75	F	COVID-19	+	97.5	BA.5.2.1	SARS-CoV-2
**NP56**	32	F	Acute respiratory failure with hypoxia, COVID-19, pelvic pain, nausea, vomiting	+	100.0	BA.5.2.21	SARS-CoV-2
**NP58**	91	F	Delirium and Non-ST-Elevation Myocardial Infarction, acute right-sided weakness, acute hypoxemic respiratory failure, sepsis with encephalopathy	+	100.0	BA.2	SARS-CoV-2
**NP59**	25	F	Unknown	+	99.1	BF.5	SARS-CoV-2
**NP62**	72	F	COVID-19	+	100.0	BA.2.12.1	SARS-CoV-2
**NP63**	62	M	COVID-19, acute hypoxemic respiratory failure	+	100.0	BA.5.2.1	SARS-CoV-2
**NP64**	19	M	COVID-19, nausea, vomiting and diarrhea.	+	100.0	BA.4.1	SARS-CoV-2
**NP68**	82	F	Acute Respiratory failure with hypoxia, elevated troponin I level, longstanding persistent atrial fibrillation, acute diastolic heart failure, weakness, non-healing wound on right lower extremity, hearing loss	−	0.0	N/A	-
**NP69**	74	M	Diarrhea, unspecified type, abdominal cramping	−	0.0	N/A	-
**NP70**	33	F	Upper gastrointestinal bleed	−	0.0	N/A	-
**NP72**	15	F	Dehydration	−	0.0	N/A	-
**NP73**	69	F	Dyspnea, unspecified type	−	0.0	N/A	-
**NP74**	0.4	F	Viral gastroenteritis, vomiting, diarrhea	−	0.0	N/A	-
**NP77**	34	F	Fentanyl use disorder, moderate	−	0.0	N/A	-
**NP78**	50	F	Bacterial upper respiratory infection	−	0.0	N/A	-
**NP80**	44	M	Polysubstance dependence, including opioid drugs with daily use, open wounds of both lower extremities, and sleep apnea	−	0.0	N/A	-

* SARS-CoV-2 diagnostic PCR with the Cepheid system. ^#^ Percentage of the SARS-CoV-2 genome covered. ^@^ Genetic lineage (Pangolin lineage) determined using the PANGOLIN v.4.3.1 software. ^ Pathogenic virus identified by agnostic sequencing.

**Table 2:** Estimated expression change, statistical significance, and function of selected genes, including the top 20 upregulated DEGs, plus *IFIH1*, *TLR3*, *MX1*, and the housekeeping gene *RPP30*.

DGE FC Rank	Gene	Fold Change	−log_10_P	Gene Product Function/ Brief Description	References
1	*CXCL11*	75.06	7.39	Cytokine (chemokine), positive chemotaxis of T lymphocytes	[43]
2	*OASL*	70.03	11.91	Oligoadenylate Synthetase-Like enhances the RIG-I pathway	[44]
3	*IFIT1*	56.1	17.73	Binds to RNA with 5′ triphosphates (uncapped), inhibiting viral replication	[45]
4	*IFIT2*	35.02	12.12	Binds to mRNAs to inhibit translation	[45]
5	*ISG15*	26.72	8.90	Binds covalently (“ISGylation”) to the viral RNA sensor MDA5, to activate it, and RIG-1 to downregulate it	[46,47]
6	*XAF1*	24.76	9.00	Interferon-stimulated gene induces apoptosis	[48]
7	*AQP9*	21.86	4.45	Aquaporin channel	[49]
8	*CXCL9*	21.41	3.96	Cytokine (chemokine), positive chemotaxis of immune cells	[43]
9	*HERC6*	19.16	10.37	ISG, E3 ISG/Ubiquitin Ligase for indirectly modulating STING	[50]
10	*DDX60L*	18.64	7.79	RNA helicase inhibiting viral replication	[51]
11	*IDO1*	18	5.86	Indoleamine dioxygenase is involved in inflammation and cancer	[52]
12	*TOR1B*	17.88	3.85	Putative chaperone for the integrity of the ER and nuclear envelope	[53]
13	*RSAD2*	17.39	9.41	Viperin, a radicalSAM enzyme, inhibitor of the viral life cycle	[54,55,56]
14	*RIG1*	17.15	7.21	Retinoic acid inducible gene encoding an RNA helicase involved in viral RNA recognition	[57,58]
15	*CXCL10*	16.8	2.94	Cytokine (chemokine), positive chemotaxis of immune cells	[43]
16	*FYB1*	15.89	4.07	FYN-binding protein 1/ADAP protein inhibits ISGylation of RIG-1 by viruses	[59]
17	*PNPT1*	15.45	6.19	RNAase is involved in the degradation of oxidized mitochondrial RNAs, virus upregulated	[60]
18	*IFIT3*	14.93	8.03	Central scaffold subunit of the antiviral IFT1/2/3 complex	[45]
19	*GIMAP4*	14.83	3.58	GTPase Immunity Associated protein 4	[61]
20	*FRMD3*	14.72	4.93	Regulator of epithelial cell development via the Notch pathway	[62]
24	*IFIH1/* *MDA5*	13.36	9.08	MDA5 RIG-I-Like Receptor (RLR), involved in viral RNA recognition	[58]
44	*TLR3*	8,94	3.65	Recognizes viral dsRNA and induces innate immunity	[63]
50	*MX1*	7.73	10.66	MxA protein sequestrates virus factors, a proposed biomarker for viral infection	[13,64]
N/A	*RPP30*	0.93	0.05	RNAseP subunit/housekeeping RNA	[38]

## Data Availability

The RNA expression raw sequencing data is available at the NCBI BioProject database as PRJNA1372799, with SAMN53806727-SAMN53806756 as accession numbers for the individual samples. The SARS-CoV-2 amplicon raw sequencing data is available at the NCBI BioProject database as PRJNA1390822, with SAMN54204617-SAMN54204636 as accession numbers for the individual samples.

## Supplementary Materials

The following supporting information can be downloaded at: https://www.mdpi.com/article/10.3390/genes17050497/s1, Figure S1: WikiPathways enriched in NP swab samples of SARS-CoV-2 infected individuals; title; Figure S2: UniProt Annotated Keywords enriched in NP swab samples of SARS-CoV-2 infected individuals; Table S1: Estimated expression change and statistical significance of genes included in this study; Table S2: Estimated expression change and statistical significance of statistically significant DEGs.
