# Supplementary figures and images for: Differential Gene Expression in Human Upper Respiratory Tract Samples Identifies Antiviral Responses in Omicron SARS-CoV-2 Infection
Source: Genes (Basel). 2026 Apr 22;17(5):497. doi: 10.3390/genes17050497 (PMC13206619; doi:10.3390/genes17050497)

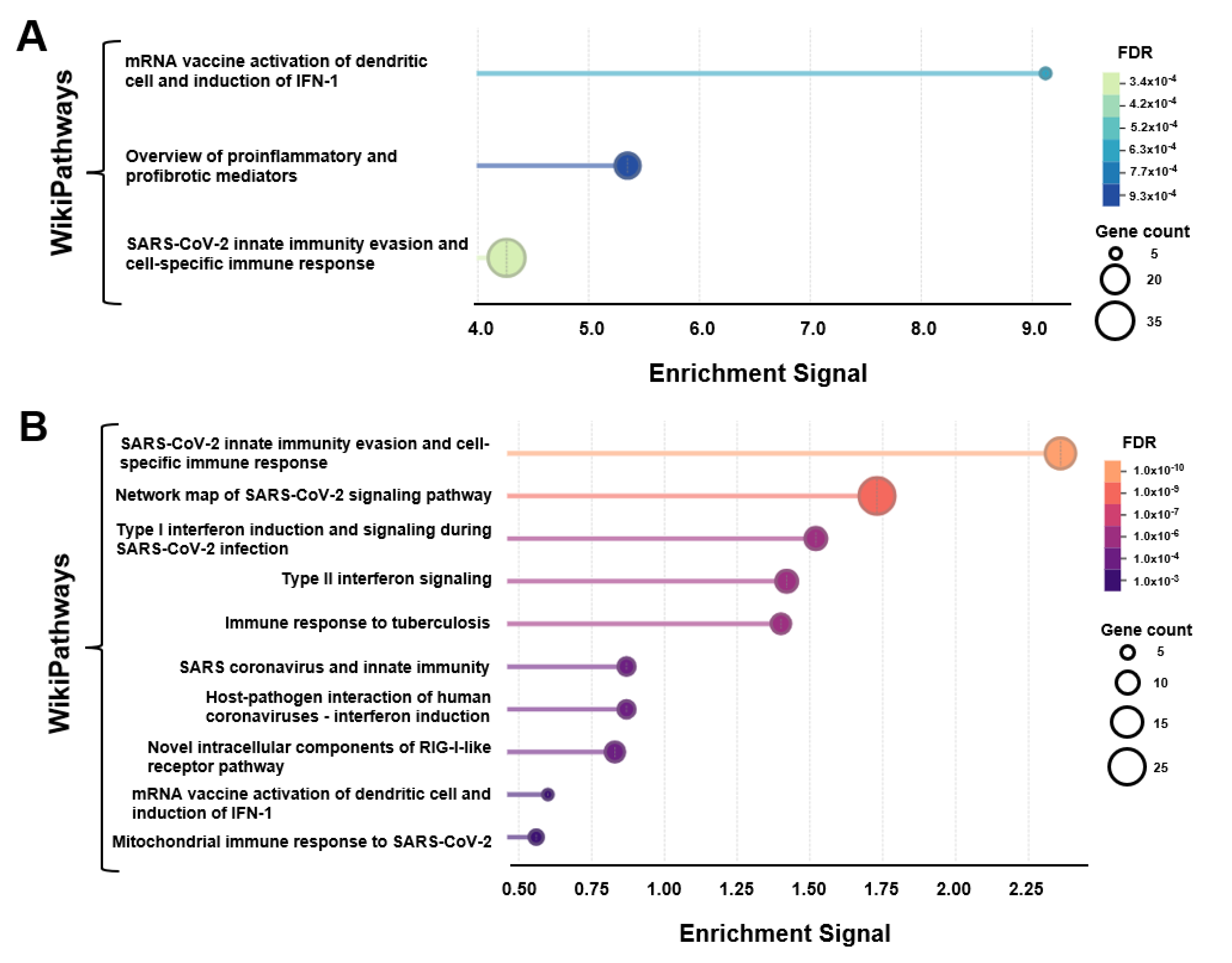

Supplement: Supplementary file 1 [file genes-17-00497-s001.zip › Supplementary Files/Figure S1.tif]

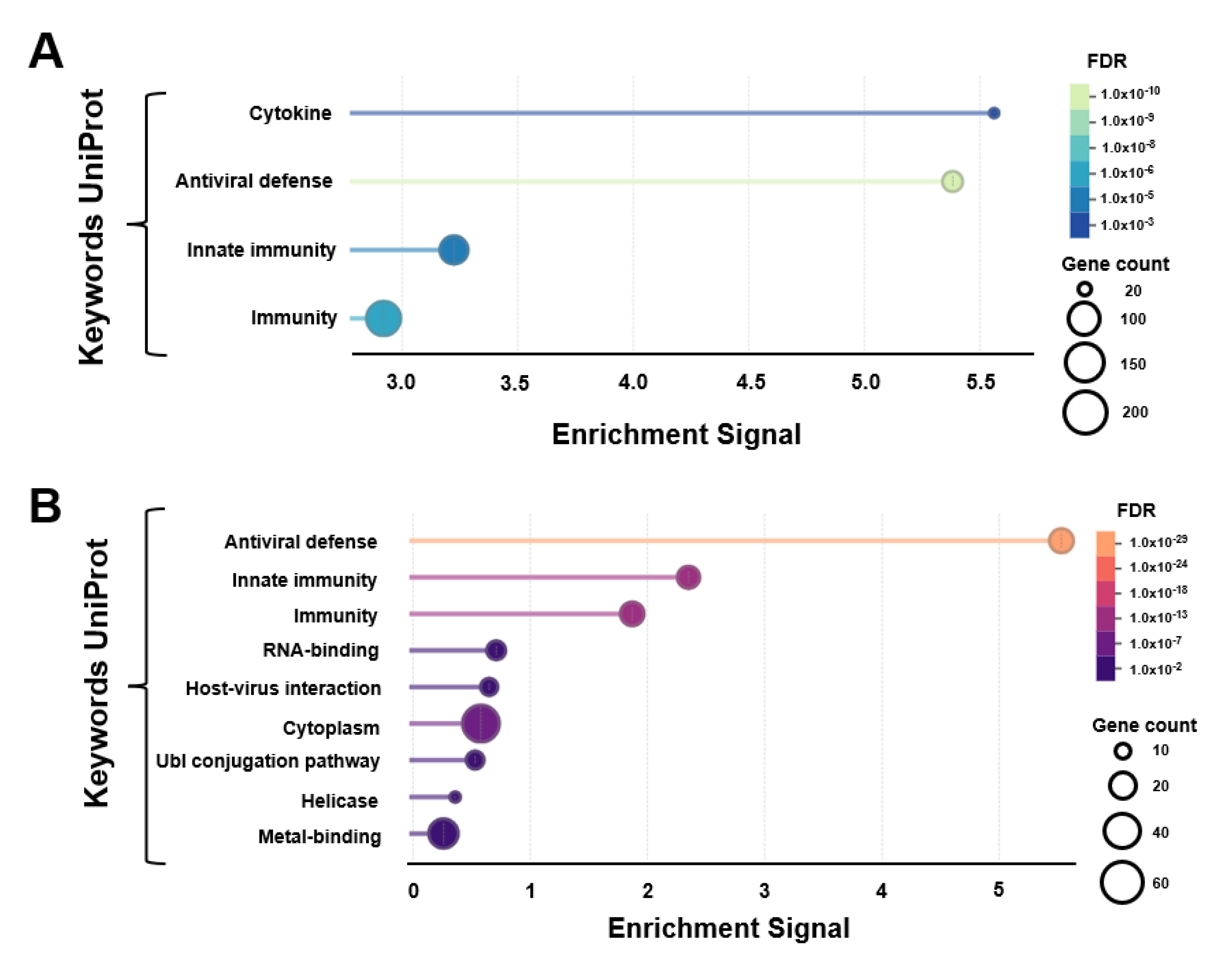

Supplement: Supplementary file 1 [file genes-17-00497-s001.zip › Supplementary Files/Figure S2.tif]
